# End-of-life care communications and shared decision-making in Norwegian nursing homes - experiences and perspectives of patients and relatives

**DOI:** 10.1186/s12877-015-0096-y

**Published:** 2015-08-19

**Authors:** Elisabeth Gjerberg, Lillian Lillemoen, Reidun Førde, Reidar Pedersen

**Affiliations:** Centre for Medical Ethics, University of Oslo, P.b 1130 Blindern, 0318 Oslo, Norway

**Keywords:** Nursing homes, End-of-life care communication, Shared decision-making, Qualitative study

## Abstract

**Background:**

Involving nursing home patients and their relatives in end-of-life care conversations and treatment decisions has recently gained increased importance in several Western countries. However, there is little knowledge about how the patients themselves and their next-of-kin look upon involvement in end-of-life care decisions. The purpose of this paper is to explore nursing home patients’ and next-of-kin’s experiences with- and perspectives on end-of-life care conversations, information and shared decision-making.

**Methods:**

The study has a qualitative and explorative design, based on a combination of individual interviews with 35 patients living in six nursing homes and seven focus group interviews with 33 relatives. The data was analysed applying a “bricolage” approach”. Participation was based on informed consent, and the study was approved by the Regional Committees for Medical and Health Research Ethics.

**Results:**

Few patients and relatives had participated in conversations about end-of-life care. Most relatives wanted such conversations, while the patients’ opinions varied. With some exceptions, patients and relatives wanted to be informed about the patient’s health condition. The majority wanted to be involved in the decision-making process, but leave the final decisions to the health professionals. Among the patients, the opinion varied; some patients wanted to leave the decisions more or less completely to the nursing home staff. Conversations about end-of-life care issues are emotionally challenging, and very few patients had discussed these questions with their family. The relatives’ opinions of the patient’s preferences were mainly based on assumptions; they had seldom talked about this explicitly. Both patients and relatives wanted the staff to raise these questions.

**Conclusion:**

Nursing home staff should initiate conversations about preferences for end-of-life care, assisting patients and relatives in talking about these issues, while at the same time being sensitive to the diversity in opinions and the timing for such conversations. As the popularity of advance care planning increases in many Western countries, discussions of patients’ and relatives’ perspectives will be of great interest to a broader audience.

## Background

As in most Western countries, Norwegian nursing home residents are old, frail and have multiple chronic diseases with dementia as the most frequent diagnosis [1]. Over the past decades, nursing homes have increasingly become the site of death, and in 2013 approximately 47 % of all deaths in Norway occurred in nursing homes [2], which is more than in other European countries [3] and the US [4].

Due to the high number of critical events and deaths, in addition to the widespread and gradual deterioration of cognitive function, it is a challenge to ensure that the period leading up to the end-of-life is in accordance with the nursing home patient’s preferences and values. This applies to what the patient wants and/or worries about in the near future, and future health care and preferences for the end of their life. Thus, the nursing home staff’s ability to appropriately deal with these questions is very important.

Eliciting the patient’s values and preference for end-of-life care and shared decision-making are central elements of what is called advance care planning (ACP), a process in which anticipatory decisions are derived through open discussions between health-care professionals, patients and/or relatives [5]. The aim is to promote future care that reflects the patient’s preferences and values, and should be offered when the patient is still able to participate in the discussion [6]. Advance care planning is seen as an opportunity to optimize care, promote autonomy and empower patients [7]. Implementing different types of advance care planning has recently gained an increased importance in several Western countries.

Over the last few decades, several studies on ACP have been conducted that focus on various perspectives, and within different settings. Various systematic reviews have recently also been carried out, which look at different issues/aspects related to ACP. Regarding the effectiveness of ACP, studies have found that ACP improves the documentation of the patient’s preferences and changes in health-care utilization, e.g. reduced hospitalization and an increased use of hospice service [8]. The meta-analysis of Houben et al. [9] found that ACP interventions increased the number of ACP discussions, as well as the concordance between patient preferences and provided care. Moreover, a review by Brinkman–Stoppelenburg et al. [10] found that ACP positively impacts the quality of end-of-life care, and that complex, process-oriented interventions were better in meeting the patient’s preferences than written documents alone.

Looking at the attitudes to discussions about end-of-life care with frail and older individuals, a review by Sharp et al. [11] found that the majority of frail older individuals would appreciate the chance to discuss end-of-life care, yet most of them have not had this opportunity. Their preferences for the timing of these discussions were highly variable. Another recent review [12] focuses on factors associated with the initiation of ACP regarding end-of-life issues in dementia, concluding that health-care staff should initiate ACP early, as long as they carefully consider the timing and the patient’s and relative’s approachability.

A study from 2007 indicated that the practices and routines for end-of-life communication only occur infrequently in Norwegian nursing homes [13], although some nursing homes have positive experiences with such communication [14]. Nonetheless, we know little about how patients and their relatives experience end-of-life care conversations in nursing homes, and their attitudes toward talking about preferences or participating in end-of-life care decisions.

In 2010, we carried out a study on existential issues and shared decision-making in a broad sense, asking nursing home patients and relatives about their experiences with the nursing home, to what extent they had been involved in conversations about their values and preferences, about participation in different kinds of decision-making and their attitudes to such questions. The study also included questions on experiences with- and attitudes to the use of coercion [15].

The purpose of this paper is limited to describing: 1) to what extent nursing home patients and relatives have been involved in end-of-life care discussions, and the desirability of such conversations, 2) their views on medical information, and 3) how they perceive their own role in future end-of-life care decisions.

At the end of 2014, people aged 80 years or more represented 4.3 % of the total Norwegian population. Approximately 18 % of these are living in institutions,^1^ while the rate of hospital admissions is more than twice as high as for other inhabitants of the community [16]. Most Norwegian nursing homes are publicly funded and operated by Norwegian municipal authorities, but roughly 10 % are private (both non-profit and commercial).

Good care at the end of life that promotes patients’ values and preferences is primarily determined by the staff’s knowledge, skills and attitudes, but the legal conditions in each country may also affect how these processes are carried out. However, the legislation in this field, e.g. concerning advance directive, varies across borders [17].

### The legal framework in Norway – patients’ rights

As in several Western countries, patients’ involvement in medical decisions has also become an increasingly important part of health policy in Norway. In Norway, the provision of the patient’s right to decide is incorporated in the Patients- and Users Rights Act [18], which states that as a main rule health care can only be provided with the patient’s consent unless a legal authority exists, or there are other valid grounds for providing health care without consent. In order for the consent to be valid, three criteria have to be met: 1) the patient must have received the necessary information concerning her/his health condition and the content of the health care, including an understanding of the consequences for her/him, 2) that she/he has the capacity to consent, and 3) the consent must be given voluntarily. Even so, when providing health care to nursing home patients, it is sometimes impossible to satisfy all three requirements in order to obtain a valid consent. According to the law, providing the patient with the necessary information is important to help make the consent valid. But who is to decide what constitutes the necessary information? Information on health conditions and future prospects may include “bad news”, and be harmful if the amount of information or speed of delivery goes beyond what the patient wants at that time. As the illness advances and death approaches, many patients may prefer less detailed information [19, 20].

From a moral point of view, an essential step in the process of giving information and facilitating decisions is to discover the patient’s current preferences for both information and an involvement in decisions. Each patient is unique and may wish for varying amounts of information at various times. Hence, the staff has to adjust the information to the patient’s current preferences and condition.

Approximately 60–80 % of the patients in Norwegian nursing homes have some kind of cognitive impairment [1, 21], a condition that may affect their ability to give informed consent, although some may still have the capacity to consent, at least for some decisions.

According to Norwegian law, when the patient lacks a competence to consent, the responsibility to decide is entrusted to the health-care professionals. The relatives of these patients have a legal right to receive the necessary information about the patient’s medical condition, and to be asked about their knowledge concerning what the patient would have wanted.

### Family involvement

The ongoing deterioration of the condition of the person with dementia makes the collaboration between family representatives and the nursing home staff increasingly important. Good information, communication and relationships between the staff and the relatives may help to facilitate shared decision-making [22]. In a qualitative metasynthesis of family involvement in decision-making for people with dementia in residential care, Petriwskyj et al. [22] found several studies that highlight trust as being highly important in family involvement in decision-making. A climate of trust exists when the next-of-kin perceive that the staff is acting in the best interest of the patient. This kind of trust is mostly built through interaction with the staff [23, 24].

Several studies have shown that relatives have a varying amount of knowledge of the patient’s values for end-of-life care [22, 25–27]. For instance, Black et al. [25] found that surrogates’ knowledge about their relatives’ wishes for end-of-life care ranged from clear instructions, to nonspecific information to no information at all. As a result, decisions may correlate more with the surrogate’s own preferences, rather than the preferences of incapable patients [28]. To help ensure that the decisions are in accordance with what the patient would most likely have preferred, it is of great importance to explore what the relatives really know about the patients’ preferences and values.

Correspondingly, we wanted to explore what the next-of-kin know about the patient’s preferences and desires for end-of-life care; had they explicitly talked about this, or were their opinions merely based on impressions and more or less indirect communication? Furthermore, have the patient’s views and perspectives been discussed with the nursing home staff, i.e. had the patients and/or their relatives participated in any conversation about the patient’s values and preferences, particularly in relation to end-of-life care issues? We also wanted to know to what extent the patient and relatives wanted to be involved in decision-making regarding end-of-life care issues, and what type of information they wanted with regard to the patient’s health condition, medical treatment and care.

## Methods

The study has a qualitative and explorative design, based on a combination of individual interviews with nursing home patients and focus group interviews with relatives.

### Sample

Six nursing homes in four different municipalities in the Southeast part of Norway were asked to participate in the study. This convenient sample of nursing homes was selected based on previous knowledge from other research projects or because they have been involved in some kind of ethics work. On average, the nursing homes had 68 beds (range 53–88), and each nursing home was asked to purposefully recruit patients and relatives who might be interested in participating in the study. The nursing home staff gave a verbal description of the study to patients and relatives, and handed out a cover letter with the same information.

#### The patients

To be included in the study, patients had to be competent to consent and be able to talk about what was important to them as residents in the nursing home. 38 nursing home residents were interviewed. We do not know how many patients were approached and how many refused to participate, but according to the staff it was easy to recruit patients for the interviews. Nevertheless, the decision capacity of two of the recruited residents was questionable, i.e. during the first part of the interview we realized that they were more cognitively impaired than expected, and the data from these interviews is not included in the analysis. One resident changed her mind, and decided not to participate. Hence, the study sample consists of 35 patients, 27 women and 8 men. The age range of the participants was 68–98 years old (mean 86 years).

#### The next of kin

Seven focus group interviews were undertaken, consisting of three to eight persons, in total 33 participants. In one of the six nursing homes, it was not possible to establish a focus group, i.e., in this nursing home we only carried out interviews with the patients. In two nursing homes, we completed two focus group interviews, whereas in the other three nursing homes we conducted one focus group interview in each of them. To be selected for focus group interviews, the persons had to be relatives of nursing home patients without the capacity to consent, and to be motivated to talk about their experiences and views as relatives to a nursing home patient. Most of the relatives were daughters, but some sons, spouses and one niece also participated in the interviews. The majority had been relatives in the nursing home for two or more years.

### Interviews

#### Interviews with patients

**Fig. 1:**
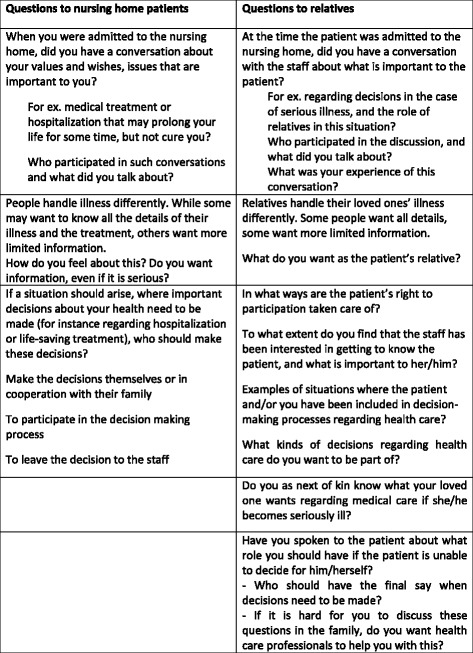
Questions used in individual interviews and focusgroup interviews, with nursing home patients and relatives respectively

A semi-structured interview guide was used as a starting point for the interviews. The interview opened with a question on how they enjoyed the nursing home and progressed to more specific questions, of which questions on end-of-life care communications, information and shared decision-making are in focus in this paper (Fig. 1). The patients were also asked about what was important to them, if there was anything they were dissatisfied with or worried about and their views on the use of coercion [29].^2^

The participants were invited to expand on the issues, describing their experiences and views. Two of the authors (EG and LL) conducted the first 10 interviews together, while the rest were divided between the two. All the interviews were carried out in the patient’s room, and each interview lasted between 20 and 40 min.

#### Focus group interviews

The interview guide covered eight questions, of which five were related to end-of-life care communication, information and shared decision-making (Fig. 1).^3^ By using focus group interviews, we aimed to develop insight into the relatives’ experiences and views through group discussions [30]. Focus group interviews are usually conducted by a single moderator, who ensures that the subjects in focus are discussed, and that all the participants are taking part in the discussion. We chose to have two moderators (EG and LL), who ran the focus group interviews together, thereby supplementing each other with follow-up questions. The relatives were encouraged to be as concrete as possible, while at the same time attempting to cover the privacy of the patient. However, that was not always possible, especially when the patients they represented lived at the same nursing home ward. The interviews lasted for about one and a half to two hours each.

### Data analysis

With one exception, all the interviews with the patients and relatives were audio recorded with consent from the participants and transcribed verbatim. Due to a damaged recorder, written notes were taken in the one interview that was not audio recorded, and the analysis was carried out in several steps. First, all four authors did a naïve reading of the transcripts and discussed the content, both during and after completing all the interviews. This gave us a first impression of the data material as a whole, but also of the answers to the specific questions.

Two of the authors (LL and EG) collaborated on a further analysis of the text. After reading through the entire material, data relating to end-of-life communication, information and decision-making were selected and re-read several times. Each of the two authors independently made a rough outline of what they found important in relation to the research questions. This formed the initial structuring of the text in specific content areas [31], and ensured an intersubjective understanding [32]. Through discussions, we agreed upon the most prominent categories in the text, and to secure the connection between the underlying text and the categories, the transcripts were read several times and compared with the categories. Answers are paraphrased in the text and presented in the form of quotations when considered typical of the respective categories. We also switched back and forth between the selected fractions of the text and the full interviews, looking for special themes that ran through the data [33].

The analyses of the data have been discussed by all four authors, and we have also presented the material and our impressions to colleagues. In addition to the research questions, the reading of literature on ACP, theories on ethical principles such as autonomy and trust, and the regulation of patient’s rights have also inspired the analyses. This is a sort of “ad-hoc” analysis [34, 35], an eclectic form of creating meaning in qualitative texts, also called bricolage [35].

### Ethical considerations

Basic ethical principles for research ethics were followed [36, 37]. All participants gave informed consent after having received written and oral information about the project. Interviewing older, vulnerable people about sensitive topics involves ethical and methodological challenges [38, 39]. In all the interviews we took time to give verbal explanations of the project, and the interviews started with simple questions aiming at enhancing a trusting relationship. Our use of the interview guide was flexible, and if the interview led to fatigue or stress, we moved to another question or shortened the interview. Participant and patient anonymity is preserved in the text. The study was approved by the Regional Committees for Medical and Health Research Ethics (REC –South East B, approval June 30, 2009, project number S-09261b 2009/6215).

The researchers who conducted the study were financed by the University of Oslo, through funding from the Ministry of Health and Care Services.

## Results

With the exception of one patient, all others agreed to participate in the study. Several next-of-kin spontaneously expressed that they appreciated being asked to participate in the focus group interview, thus giving them an opportunity to talk about issues they seldom discussed. The patients’ answers were often shorter than expected, and their ability to delve into these issues seemed a bit reduced, possibly because the questions might have been found to be complicated and emotionally challenging.

Three main categories emerged from the interviews, and in the following the answers from patients and next-of-kin are presented separately:

- The lack of end-of-life care communication
- Wishing to be informed
- Shared decision-making

### The lack of end-of-life care communication

#### Patients

Very few patients explicitly said that they had participated in a conversation, either a short time before they were admitted to the nursing home or a few days after the admission. However, for most of them, the content of this discussion had primarily been about practical issues, e.g. who was their nearest relative.

Most patients stated that they had not had an opportunity to discuss their values and preferences for treatment and care related to end-of-life with the nursing home staff. The following quotations illustrate the experiences of several patients:

*“No, nothing serious like that, but they ask me things like, am I happy here, is everything ok?”*

*“I think that what has been missing everywhere is an introduction. Something that tells you what is expected of you, what you can expect of them … No, nothing…. Nothing that has to do with my personality, no.”*

Even so, some used the expression, “I can’t remember.” That is, they could not guarantee whether they had or had not talked about this, but they could not recall such conversations.

The patients were also asked about the desirability of such conversations, and their answers can be divided into two groups: Those who wanted such conversations and those who did not see the necessity for such discussions.

#### Those who wanted such conversations

Some of those who said they had not participated in a conversation about future preferences for medical treatment and care explicitly said that they wanted or missed this kind of conversation:

*“Maybe that’s precisely what is missing … Actually I do (miss this sort of conversation).”*

*“But I would’ve been happy for it. What I would do, and what I want help with and such. I would’ve liked to talked to them more about it.”*

More or less explicitly, they expressed feelings of not being recognized as the person they are, with specific needs and preferences, and that the occurrence of these conversations was left to chance:

*“No, I don’t think they do (have knowledge of her values and preferences). They don’t have time for it. But sometimes they do sit down, and we can discuss things I have been thinking about.”*

According to some patients, the staff’s lack of knowledge of the patient’s values and preferences was mostly caused by their own introvert personality; they did not easily talk about what is important to them:

*“I don’t think…, I mean, I haven’t encouraged it.”*

*“I’m not exactly outgoing.”*

#### Such conversations are not necessary

Nonetheless, other patients expressed that there was no need for a special conversation about their wants and preferences for end-of-life care in the future. They assumed that their wishes were known and that the staff knew what was important to them, thus expressing great confidence in the staff’s intention to act in their best interest in the case of deteriorating health. Patients who did not miss this kind of conversation said in different ways that if they wanted to, they could talk to the staff any time. They did not feel the necessity for a specific discussion about preferences and wishes for the end-of-life. As illustrated by the following quotation, the answers were frequently a bit evasive or shifted to a more practical direction:

*“No, I don’t need it. Whatever you want, they do it for you.”*

#### When their health deteriorates …

In a follow-up question, which explicitly asked the patients about what they would want if their health worsened, several said that they did not want to prolong their life:

*I’m thinking that if I took a turn for the worse, and am this old (94 years old), it would be nice to just be allowed to let go in a peaceful way. Without pain and such. I think, well, that’s life…. And it has to end…. I don’t want treatment if I have an illness that is just going to be prolonged.”*

Asking her if the staff knew about her views, she said*: “No, I don’t believe they do.”*

This lady was one of several who doubted that the nursing home staff knew their preferences for end-of-life care. With some exceptions, most patients had neither talked with their family about their preferences for end-of-life care, nor had they discussed what kind of role their relatives should have in the decision-making process if they became seriously ill and were unable to make their own decisions.

#### Next-of-kin

Very few relatives had participated in conversations about what was important to the patient and her/his preferences for end-of-life care. But as reported by the patients, most of these conversations had primarily been about practical issues:

*“I went to a meeting when we first got here. But it was mainly practical, technical, yes – it was very personal, but more existential questions were not brought up. On the other hand, she (the patient) had an incident and was admitted, and in the aftermath we (he and the doctor) talked about these things.”*

The quotation illustrates an experience that several relatives had: that the staff did not initiate a discussion with the family until the patient’s health had severely deteriorated:

*“We didn’t have that kind of conversation when she arrived here, but we had one later because mom got very sick…”*

Some relatives had themselves initiated this kind of conversation:

*“Hospital admittance was not brought up much, but I have talked to them about prolonging life. I don’t want that. You shouldn’t start all kinds of things just to keep going artificially. I said that I wanted to inform them of that.”*

Irrespective of the experience with this type of conversation, who had taken the initiative or what had given rise to it, most relatives expressed that they wanted a conversation about the patient’s wants and preferences for end-of-life care, even when such conversations might be emotionally difficult. However, they would appreciate that the staff took the initiative, but stressed that it should take place at “the right time”.

#### Relatives’ attitudes to the timing of end-of-life care conversations

The timing of this type of conversation was an important issue raised by several relatives. In discussing “the right time” for such conversations, the relatives exposed a great variety of opinions. Several wanted to postpone this type of conversation, expressing that talking about these issues should wait until the patient’s health deteriorates:

*“I assume that the question will come the day they get sick, that we will be included then.”*

In this lies an expectation that the staff would initiate this kind of conversation when "the time comes", and that this was ok by them.

Some relatives had found the admission to the nursing home to be emotionally difficult. To be involved in an early conversation about what to do when the patient’s health deteriorates would have been too much for them:

*“That would’ve been difficult for me. First, you are arriving at your final destination, you know you are, and then to start speaking of death. I wouldn’t want to.”*

Some said explicitly that such questions were too difficult to talk about at all, especially in the present situation, since their loved one “was not that sick.”

Another reason for postponing this conversation was related to changes in the patient’s condition during the stay; the patient’s opinions may change, and thus it may be a bit premature to talk about these questions at admission to the nursing home or shortly after.

Still, some relatives claimed that such conversations should be carried out as soon as possible:

*“These are really important questions, so they should be brought up right away.”*

They feared that postponing these conversations could have negative consequences if the patient’s health condition deteriorated rapidly. It could be too late if you wait until the patient’s health goes from bad to worse. They thought that the benefits of early discussions outweighed any discomfort, as they wanted to be prepared:

*“I want to think it through now, and prepare myself. That’s what I thought.”*

### Wishing to be informed

#### Patients

Most patients were satisfied with the information that the health care staff provided about their health condition. Nevertheless, some found the information to be insufficient and coincidental. The importance of the physician’s role in informing them about their health condition was mentioned in particular, and some claimed that they seldom saw the physician.

Most patients wanted to be fully informed about their health condition, including information of a serious nature, for example revealing a severe deterioration of their health condition:

*“If I take a turn for the worse, I want to know. Even if it’s serious.”*

*“Of course I want to know as much as possible, so I can handle my own illness.”*

However, some patients said that they had not reflected upon this issue, and they were not sure that they really wanted all the information. A minority explicitly said that they did not want to know everything, due to the possible harmful character:

*“…that will just leave me thinking. And I would rather not.”*

Or they said because they were not that interested any more, and trusted the staff to act in their best interest:

*“When you’ve almost reached 100, you’ve got to trust others.”*

#### Next-of-kin

As with most patients, the next-of-kin were, with few exceptions, very satisfied with the information they received from the staff. For example, every next-of-kin who had experienced hospitalization of the patient had been informed about the intervention, either before the decision was made, or shortly after. Nevertheless, some relatives expressed that the information was given too randomly, depending on who was on duty.

All the relatives wanted to be informed about the patient’s health condition, and of special importance was information concerning changes in their health:

*“We want to know everything. …. I want to know what is happening.”*

However, when they say “everything” they did not want all the details about the health condition and treatment, just the main features. When it comes to less serious issues, for example problems with giving medication and behavioural disorders, most of them did not want to know.

### Shared decision-making

#### Patients

Few patients had reflected upon who should decide when important decisions about medical treatment had to be made. When questioning the patients about who should be involved in such decisions, three categories of answers came up: 1) to participate in the decision-making process, but leave the final decision to the staff, 2) to leave the entire decision-making process to the staff, and 3) to have the full responsibility.

#### Participating in decision-making processes

Many patients wanted to participate in the decision-making process, although they wanted the staff to make the final decision. Yet, few had been asked about things such as hospitalization or medication when this had been in question. They emphasized that they wanted their voice to be heard:

*“Yes, I do want to be part of the decision, along with the staff…. whether or not to be admitted to the hospital. If something happens….with me, I go to the nurses for advice. And they always ask me what I think…. They have to ask me what I think.”*

*“I want to know what they are doing. But the person who knows best should decide, whoever it is that knows… I don’t know enough to decide.*

That is, they wanted to be informed and involved, but left the decisions to the medical staff, trusting their competence.

#### Leaving the decision-making process to the staff

The second group comprised those who expressed that it was okay or reasonable to leave all these decisions to the staff, hence exposing a great confidence in the staff’s intention to act in their best interests:

*“I think that’s ok (that the staff decides). The doctor was here yesterday, and he knows what’s best. I trust him. I’m nearly 100, I have to trust others.”*

*I can’t go against those with authority, they know best…. They are able to decide, not me.*

#### Having full responsibility for the decision-making

The third group comprised the very few who wanted to decide themselves, to be responsible for these kinds of decisions:

*“Those are mine to make (decisions about hospital admittance or life-saving treatment). They will have to explain why…., and I get to decide.”*

#### Next-of-kin

Most relatives wanted to be involved in decision making concerning health-care issues if the patient was no longer able to decide on their own, but did not want decision-making authority:

*“I would like to be informed and consulted, even though I respect the professionals’ knowledge. Maybe I will follow the advice, but I want to be part of the conversation.”*

Yet, there are great variations in how they wanted to be involved, e.g. in situations when decisions have to made very quickly. Some found it acceptable that the staff made the decisions, as long as they were informed afterwards:

*“But if mom should become acutely ill, I would absolutely want her to be treated right away without conferring with me.”*

On the contrary, others wanted to be involved as early as possible.

When it comes to decisions about life-prolonging treatment, most relatives wanted to be consulted, but at the same time they emphasized their respect for the complexity of these decisions, and that it is the staff who has the competence to make the proper decisions:

*“… I want to be part of the conversation, I want to participate in the discussion of medication and such, but specifically saying yes or no, I feel like that is too much responsibility for me.”*

This statement was supported by several other relatives, who expressed that they feared being personally responsible for such decisions, displaying confusion about who was responsible for the final decision:*“…We thought it was the end, and it was a very difficult process because they ask, ‘Do you want her to be hospitalized and be saved…no matter what?’ And those kinds of situations are very difficult, what are you supposed to say to that?…So I was very happy to hear that it was not my decision, because those things are the kinds of things you’d continue to think about…I am happy the doctor lets us know when it (further treatment) no longer can be justified.”*

#### Do the next-of-kin know the patient’s preferences?

The interviews revealed that relatives’ understanding of the patient’s wishes ranged from, in a few cases being well informed, to no knowledge at all. Very few had talked with the patient about their thoughts and preferences concerning life-prolonging treatment if their health should deteriorate. However, many relatives said that from knowing their mother or father over time, they had a clear sense of their preferences for life-prolonging treatment. Some said that they knew that their father/mother mostly wanted to die, but that they had not discussed what to do when decisions had to be made:

*“I know she doesn’t want to suffer. She’d rather let go than lie here for a long time in pain.”*

Many relatives expressed that talking about questions of life-prolonging treatment with their father or mother was difficult for different reasons. For instance, some emphasized that they should rely more on their knowledge from previous years than listening to what the patient was saying now due to their cognitive impairment:

*“…of course when you become the caregiver for your mother who is losing control of her life, and is no longer accountable, then you have to trust yourself, not what is said. Be confident enough that you know your mom so you can keep her best interests in mind.”*

Some recognized that the patient’s perspectives might alter as their health condition changed during their nursing home stay. For example, one of the relatives said that some years ago his mother had signed a “living will” document, expressing that she did not want any kind of life-prolonging treatment. However, he now found that she did not want to talk about this, and he was asking himself if she was not that sure any longer?

## Discussion

Very few nursing home patients and relatives had participated in conversations with the nursing home staff about preferences and wishes for end-of-life care. Some patients wanted to talk about this, while others were reluctant or indifferent to such discussions. Although most patients wanted their voice to be heard in decisions about medical treatment, several patients wanted to leave the decisions to the health-care professionals.

Most of the relatives wanted to take part in conversations about end-of-life care, but at the “right time”. Although most relatives wanted to be involved in important decisions about treatment and care, they entrusted the staff to make the final decisions.

### Limitations

The selection of institutions was based on our knowledge from previous research projects. As a result, these nursing homes may not be representative. Moreover, we only interviewed relatives of patients with cognitive impairment. This is a group of relatives who have some experiences that may differ from the relatives of patients who were cognitively well-functioning, which could have affected the results. Therefore, the results were not transferable to all the next-of-kin in nursing homes. We also could have interviewed the patient and his/her nearest relative together. This could have given us more and richer data, but at the same time this might have initiated a conversation about sensitive topics that they were not prepared for, which may have influenced each other’s replies.

The following discussion will focus on three main topics:

1. The variations in opinions and wishes for end-of-life care conversations;
2. How to understand the pervasive trust that patients and the next-of-kin have in the staff’s ability and willingness to make decisions in the patient’s best interest;
3. Information and shared decision-making.

### Variations in opinions and wishes for end-of-life care conversations

Very few patients and relatives had participated in any type of conversation about end-of-life care. Although some patients had certain reservations with regard to what they could recall, their answers were in accordance with the answers of the next-of-kin. But while most relatives wanted such conversations, the interviews demonstrated that although some patients were positive about talking about these issues, others did not find it necessary or were more reluctant. Some of these were convinced that the staff already knew their preferences and would therefore act in their best interest, whereas others signalled quite the opposite; i.e. they doubted that the staff knew them.

Previous literature provides a varied picture of what patients want. A review published in 2013 [11] reported that a majority of older individuals (61–91 %) wanted to discuss their end-of-life care. However, some of the included papers found a reluctance to have such discussions. This supports the findings in our study, namely that not all patients felt it essential to have specific conversations about this subject. Some may find it difficult to predict their future experience of illness, while others just wanted to leave the subject “in peace”. Their willingness to engage in such discussions may change over time, thus it is important to re-offer a discussion at a later stage. The low prevalence of such conversations may also be explained by the health-care professionals’ feelings of uneasiness towards discussing end-of-life care issues [40, 41]. In our study, the next-of-kin were especially explicit on wanting the staff to raise the matter, but the desire for the staff to initiate a conversation about these issues was also more or less implicit in the interviews with the patients. If the staff did not help them, the patients’ and relatives’ needs may remain unmet.

Even though most relatives would like to participate in conversations about end-of-life care, very few wanted such discussions in connection with- or shortly after the admittance to the nursing home. They feared that it could be too much of a strain, both to the patient and themselves, and emphasized the importance of “the right time”. Data on the best timing of conversations about end-of-life care is conflicting [6]. The staff has to be sensitive to the patients’ and relatives’ receptiveness or reluctance to discuss these topics [12], thereby indicating that the communication skills of health professionals are a crucial factor. A component of such highly skilled communication is to know when not to proceed with the discussion, and how to spread information over time. Caution in discussions is obviously needed.

According to the literature, the evidence regarding the benefit of discussing plans for end-of-life care with patients is mixed [6]. On the one side, patients can find the process itself helpful, particularly when discussions focus on their goals, values and beliefs, rather than on specific interventions, which may help them feel acknowledged as individuals by the nursing home staff. There may also be other important benefits, including receiving care that is aligned with their wishes [9, 42, 43], and improving documentation of the patient’s preferences [8]. Advance care planning is also thought to help families to prepare for the death of their loved one [44]. On the other side, there are some risks and barriers one has to keep in mind. Besides feelings of uneasiness, some patients may also feel that they do not have sufficient information to engage in such discussions, related to an uncertainty concerning their future illness and decline. Other barriers include the reluctance of family members and/or the staff to discuss end-of-life care, as well as time restraints for the staff [5, 11].

Several Western countries have introduced different forms of “living will” documents, “advance directives” and processes of advance care planning as methods to safeguard the patient’s values and preferences. At the same time, the increased distribution of these types of documents and processes implies a normative assessment, i.e. it is beneficial to involve the patients and relatives in such discussions. The results from our study show that it cannot be taken for granted that all patients want such conversations. Respecting the person and promoting patient autonomy also means being sensitive to patients’ preferences to not talk about these issues [19].

### Trust

Trust was a theme that was more or less explicitly brought up in many interviews. Most patients and relatives expressed a great confidence in the staff’s ability to make decisions in the patient’s best interest. As demonstrated in another part of this study [15], this was also the case when asking about the use of coercion to carry out the necessary health care. As expressed by most patients and relatives, the pervasive trust in the staff’s good intentions can be understood both in light of the general level of trust in society and in the vulnerability of nursing home patients, in addition to resulting from a trusting relationship with the staff [23, 24]. Studies have revealed that confidence in societal institutions, such as the health-care system, is especially high in the Nordic countries [45]. This means that when admitted to a nursing home, elderly people probably have a basic trust in the staff’s ability to properly take care of them. Trust is particularly important for frail, elderly people, because to be in need of nursing home care implies a loss of your ability to take care of yourself. Consequently, they have to rely on the goodwill and competence of their caregivers to have their needs fulfilled.

An essential element in the very definition of trust is the firm belief that the other, the trusted one, will act in your best interest [46]. Even so, trust has to be actively cultivated in the daily relationship between patients and staff. The patients and their relatives expect the staff to have clinical skills, comprehensive knowledge and a professional attitude. In other words, the staff has to demonstrate the will and ability to get to know the patient, and to show that they are interested in their history, values and preferences [47]. The fact that some patients expressed hurt feelings, claiming that the staff did not know them, is the opposite of a trusting relationship. Building confidence seems to be an important part of the first weeks after admittance. Without a trustful relationship, the patients will probably not want to talk about such sensitive topics as preferences for end-of-life care.

A lack of trust that the staff will act according to the patient’s values and preferences has probably also been an important reason for the emphasis on patients’ rights, as well as the increasing emphasis on living wills, advance directives and advance care planning in several countries. Although the paternalistic approach was intended to act in the best interest of the patient, it fell short because the approach did not account for the patients’ views in the decision process [48]. However, although patients’ rights, including their rights to decide, have increased over the last few decades, there are still remnants of the paternalistic culture in health care. Moreover, today’s nursing home patients belong to a generation that is trained to do what the medical staff recommends, i.e. the principle of autonomy may be a bit delusive.

As patients’ rights have increased in most Western countries over past decades, it is a question whether today's young people will have the same confidence in the health-care professionals and their advice as the elderly do today. However, trust-based decision making will probably also be an important supplement to informed consent in the future.

### Information and shared decision-making

The results revealed that not all patients wanted to be fully informed about their health condition; some wanted to be protected against information of a potentially harmful character. The great variation in their answers demands that the staff be highly attentive to the patient’s wishes for different types of information, which is also in accordance with codes of ethics for Norwegian doctors and nurses [49, 50].

When asking about involvement in decision-making about end-of-life care, it was a bit surprising that very few patients had reflected upon this before the interview, nor was this subject discussed with their relatives. The focus group interviews supported this; very few relatives had talked with their loved ones about preferences for end-of-life care or what their role should be if the patient was no longer competent to decide. A presupposition for building medical decisions on family members’ information is that they know the patient’s wishes and preferences, but conversations about end-of-life care are emotionally difficult, and most patients and next-of-kin may need assistance from the staff to raise these questions.

We found great variations in the patients’ preferences for involvement in decision making. While some wanted to leave the decisions more or less completely to the nursing home staff, several wanted to be involved, to be part of a shared decision-making. A recent study on a geriatric hospital ward in Norway found that all patients wanted to be involved in the decision-making process, in cooperation with their physician [51].

An earlier study of end-of-life decisions in Norwegian nursing homes found that doctors very seldom involved patients in end-of-life care decisions, but instead consulted the next-of-kin [52], even though the patient was still competent. This is not in accordance with laws and guidelines. There may be different reasons not to involve the patient in plans and decisions for end-of-life care, including time constraints of the health professionals, a lack of confidence in initiating and leading such conversations, fear of upsetting the patient or the fact that patient’s preferences might change. Patient predictions of preferences and reactions to hypothetical future events are both inaccurate and unstable over time [53]. That is, involving patients in plans and decision for end-of-life care is not a onetime effort, but has to be a continuous process that adapts to the patient’s condition and needs.

Shared decision-making will necessarily take different forms in different situations, and does not mean the same thing in all cases and situations [54]. For this reason, it can best be understood as a continuum. At one end is patient- or proxy-driven decision-making, while at the opposite end is physician/staff-driven decision-making. This model attempts to navigate the tension between paternalism and autonomy in medical decision-making [48].

## Conclusion

Advance care planning has gained increased attention in many Western countries over the last decade. However, the results from our study reveal that advance care planning must be individualized, as not all patients see such conversations as desirable. The staff’s obligation to uphold the patient’s autonomy requires a high degree of sensitivity when asking the patients about preferences for their end-of-life care. Patients may need time and repeated conversations, and these conversations should be seen as a continuous process that is receptive to changing opinions.

Conversations about end-of-life care issues are emotionally challenging for patients, families and staff, and few relatives had discussed these questions with the patients. Thus, it is required that health-care professionals take responsibility for initiating these conversations, based on the individual patient’s need for information and involving families according to the patient’s competency to consent, thereby preparing for a shared decision-making in a trusting relationship.

## Footnotes

1 At the end of 2013, there were approximately 41,600 beds in Norwegian nursing homes, more than 2.5 times the number of hospital beds (SSB)

2 The complete interview guide can be obtained by the first author.

3 The focus group interview opened with a question on how they experience to be relatives at the nursing home, how they were taken care of by the staff, as well as positive and negative experiences. They were also asked about their attitudes toward the use of coercion, in addition to questions in focus in this paper.
